# An essential reference for the drug safety practitioner

**DOI:** 10.1186/1479-7364-7-3

**Published:** 2013-02-05

**Authors:** Andrew A Monte

**Affiliations:** 1 Department of Emergency Medicine, University of Colorado Denver, Aurora, CO, 80045, USA; 2 Department of Pharmaceutical Sciences, University of Colorado Denver, Aurora, CO, 80045, USA

**Keywords:** Book review, Cobert's manual of drug safety, Drug safety, Pharmacovigilance

## Abstract

**Book details:**

Cobert B

Cobert's Manual of Drug Safety and Pharmacovigilance: Second Edition

Sudbury: Jones & Bartlett Learning; 2012

408 pages, ISBN-13: 978–0763791599

## 

The second edition of *Cobert's Manual of Drug Safety and Pharmacovigilance* seeks to serve as manual for students and reference for practitioners in the field of drug safety. It is a lofty objective to fulfill both of these roles, and the book seems to serve best as a reference manual for those actively practicing in the field.

The manual is well organized and edited, and it includes a CD with a portable document format (pdf) version of the book. The introduction admits that ‘genomics is barely mentioned in this book’, and indeed, the book has stayed true to this promise. Ignoring this major topic in the field of drug safety and pharmacovigilance further supports the assertion that the appropriate role for this book is as a reference for the practitioner in a drug safety department rather than a new student text. The book starts by giving the obligate definitions of the terms that will be covered in the subsequent chapters. The chapters then progress logically, focusing first on issues of drug safety from clinical trials, then post-marketing vigilance, a discussion of the entities responsible for monitoring and enforcement, and then the populations affected by drug administration. The book then moves into the specifics of how the practitioner can effectively monitor and report adverse drug reactions. These chapters are where the book truly shines. Chapter 8, entitled ‘Where the Data Resides’, is an excellent overview of global data collection devices and their strengths and weaknesses. The book has excellent online references covering drug safety links, such as Motherisk and AERs, as well as regulatory websites, such as the United States Food and Drug Administration and the European Medicines Evaluation Agency. Unfortunately, the websites have not been included in the text in order to save space. This decision makes the text more readable but makes it more difficult to access the information when needed for reference. The web links are listed at the end of the book and are ‘active links’ in the pdf version on the included CD. While the book generally provides a brief synopsis of a topic with bulleted points or references, occasionally, authors over-quote regulatory documents when the reference itself would suffice or over-bullet, which makes the associated section difficult to read. Chapter 37, the chapter discussing the International Conference on Harmonization, suffers from these flaws. The topic is essential for a student who is new to the field and to a practitioner needing an easy reference, but the chapter has too little background information and is over-bulleted, making it unreadable. Contrast to this format with chapter 40, entitled ‘Organization of a Typical Drug Safety Department’, the author gives an excellent overview of the structure of a drug safety department with practical advice about how to establish such a department. Each chapter concludes with the ‘frequently asked questions’ which lends depth to the respective chapters. These questions seem to serve as thought-provoking tools for students since the chapters often lack the discussion necessary for student level learners.

Students learning the historical background, basics concepts, and future direction of the field should continue to use such books as the *Textbook of Pharmacoepidemiology* or Stephen's *Detection of New Adverse Drug Reactions*. Despite its shortcomings for a new student in the field, *Cobert's Manual of Drug Safety and Pharmacovigilance* concisely places a large amount of information essential to the daily activities of a drug safety department in one easy-to-navigate text. This book is an essential reference for those working in the field of drug safety.

## Competing interests

The author has no competing interests to declare.

## Authors’ information

AAM is an assistant professor at the Emergency Medicine and Medical Toxicology departments of Emergency Medicine and Pharmaceutical Sciences, University of Colorado Denver-Anschutz Medical Center in Aurora, CO, USA.

